# Engineering Acoustic Metamaterials for Sound Absorption: From Uniform to Gradient Structures

**DOI:** 10.1016/j.isci.2020.101110

**Published:** 2020-04-28

**Authors:** Xiuhai Zhang, Zhiguo Qu, Hui Wang

**Affiliations:** 1 Key Laboratory of Thermo-Fluid Science and Engineering of Ministry of Education, School of Energy and Power Engineering, Xi'an Jiaotong University, Xi'an 710049, P.R. China; 2 School of Aeronautics, Northwestern Polytechnical University, Xi'an 710072, P.R. China

**Keywords:** Acoustics, Materials Design, Materials Structure, Metamaterials

## Abstract

The traditional sound absorption problem has not been completely resolved over the last 200 years. At every stage, its research has changed depending on practical requirements and current technologies. Phononic crystals (PCs) and acoustic metamaterials (AMs) have gained attention because of their extensive investigation and development over the past 30 years. Especially, the use of these materials brings new vitality into the traditional sound absorption problem to figure out broad working band and low-frequency absorption. This review highlights recent progress in sound absorption—from airborne to waterborne absorption—and gradient-index AMs. Progress in gradient-index AMs is singled out because of their favorable impedance matching, good viscous and thermal dissipation, and lengthened propagation paths compared with those of other materials. The progress in sound absorption of PCs and AMs is promising to serve as the next-generation sound absorbing materials, trap and reuse acoustic energy, and attenuate earthquake/tsunami wave in the future.

## Graphical Abstract

**Figure:**
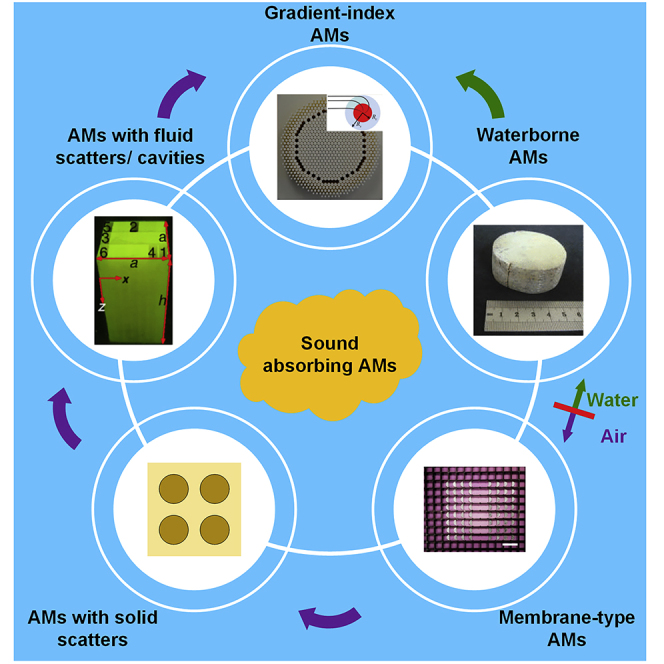

## Introduction

Metamaterials are artificially structured materials with unusual properties, which cannot be found in natural materials (Coulais et al., 2017, Page, 2011). Since Victor Veselago's seminal work demonstrating the feasibility of negative refractivity materials in 1968 (Veselago, 1968), metamaterials have attracted tremendous attention, especially in the last three decades. Metamaterial research is interdisciplinary and includes fields such as electromagnetics, optics, solid-state physics, and acoustics. Inspired by the emergence and development of photonic crystals, phononic crystals (PCs) were first proposed by Kushwaha (Kushwaha et al., 1993). Also known as sonic crystals, PCs are usually considered composites or nonuniform materials with periodic structures to manipulate acoustic wave propagation (Hussein et al., 2014, Kushwaha et al., 1993). Early research on PCs focused on the acoustic forbidden band and sound attenuation. The experiment on sound attenuation by a sculpture in 1995 gave a luminous introduction of PCs to the public (Martínez-Sala et al., 1995). On the other hand, in 2000, Liu (Liu et al., 2000) contributed their seminal article on acoustic metamaterials (AMs). AMs are usually considered artificial structures with periodic or non-periodic elements, which exhibit unusual properties beyond those of natural materials. After 2000, AMs underwent rapid development, and a plethora of novel and/or interesting AMs emerged, such as those with negative elastic modulus, negative mass density, and negative refraction as well as cloaks, mirages, metadiffusers, metasurfaces, rectifiers, and basic logic gates. It is worth noting that, in this review, the concept of AMs is extended to include PCs (Lu et al., 2009).

Moreover, AMs bring new vitality into the traditional acoustic application: sound absorption. The use of AMs offers new insights as it facilitates figuring out broad working band, low-frequency absorption, and high absorption with thin thickness. Specifically, membrane-type, cavity-based, and gradient-index AMs have enabled important contributions and inspirations in sound absorption. During the studies, the understanding of impedance matching and conversion or dissipation of energy carried by an incident wave contributes to a new generation of AMs for sound absorption. For the dissipation in AMs, AMs are usually at the subwavelength scale, whose characteristic length is smaller than the wavelength of the acoustic waves, which necessitates accounting for viscous and thermal losses (Yang and Sheng, 2017). Besides, to realize novel or specific functions, the structures of AMs are usually complicated. Thanks to the technology of additive manufacturing, the preparation of AMs with complicated structures is feasible. Because the study of AMs is an emerging discipline, just a few AM reviews have been published, and these reviews mainly focus on the fundamental concept and/or mechanics (Hussein et al., 2014, Sigalas et al., 2005) and novel and/or interesting functions (Assouar et al., 2018, Ge et al., 2018, Jin et al., 2019, Lu et al., 2009, Ma and Sheng, 2016). When it comes to sound absorption of AMs, there are only few relevant reviews. Bobrovnitskii and Tomilina (Bobrovnitskii and Tomilina, 2018) attempted to resolve the problem of sound absorption through a linear formulation. Yang and Sheng (Yang and Sheng, 2017) focused on the transition of sound absorption from porous media to AMs and presented a designed integration strategy (Yang and Sheng, 2018). However, there is no review focusing on the transition of sound absorption from common AMs to gradient-index AMs to assist the search for potential next-generation sound absorbing materials.

**Figure 1:**
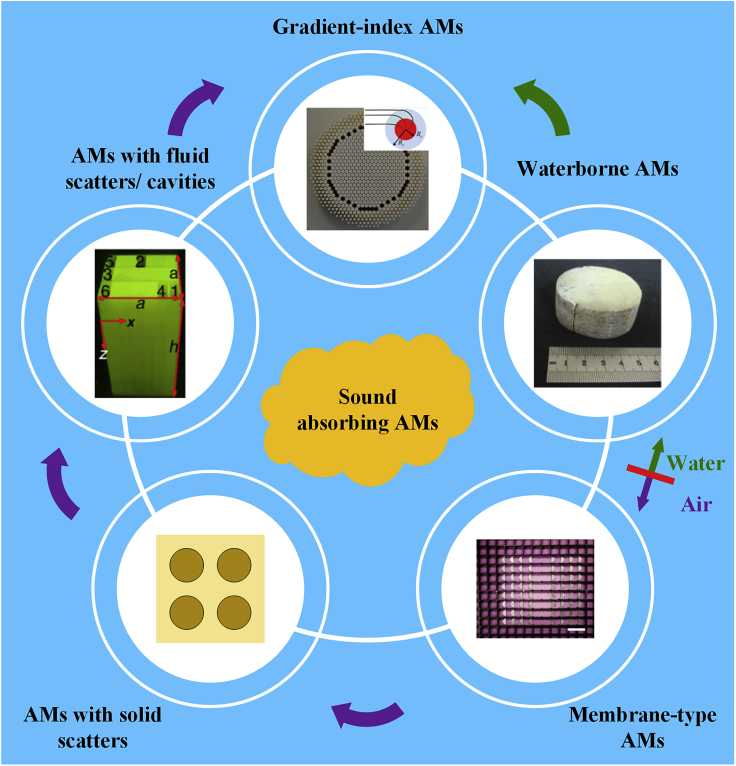
Sound Absorbing Acoustic Metamaterials (AMs) Purple and green arrows represent the development of airborne and waterborne sound absorbing AMs, respectively, with the widening of the working band. Image for membrane-type AMs is reprinted with permission from (Mei et al., 2012): Springer Nature, Copyright (2012). Image for AMs with fluid scatterers/cavities is reprinted with permission from (Zhang and Hu, 2016), Copyright (2016) by the American Physical Society. Images for waterborne AMs and gradient-index AMs are reprinted from (Jiang et al., 2009) and (Climente et al., 2012) with the permission of AIP Publishing, respectively.

This review highlights sound absorption in AMs from airborne to waterborne absorption as well as gradient-index AMs. In the section Sound Propagation through a Material, the basic concepts for sound absorption in AMs are first introduced for both researchers and beginners. Figure 1 illustrates a clear thread of the following details of this review. Considering background media, the sound absorption of AMs is classified as airborne and waterborne sound absorption, which are introduced in the sections Airborne Sound Absorbing AMs and Waterborne Sound Absorbing AMs , respectively. In the section Airborne Sound Absorbing AMs, membrane-type AMs, AMs with solid scatterers, and AMs with fluid scatterers/cavities are introduced, focusing mainly on the cavity-based AMs. The section Waterborne Sound Absorbing AMs briefly introduces the sound absorption of AMs in water. Because the impedance matching between gradient-index AMs and background media contributes to enhancing sound absorption, gradient-index AMs are investigated in detail in the section Gradient-Index Sound Absorbing AMs. The section Summary and Perspective summarizes this review and outlines some prospects for AM development.

## Sound Propagation through a Material

**Figure 2:**
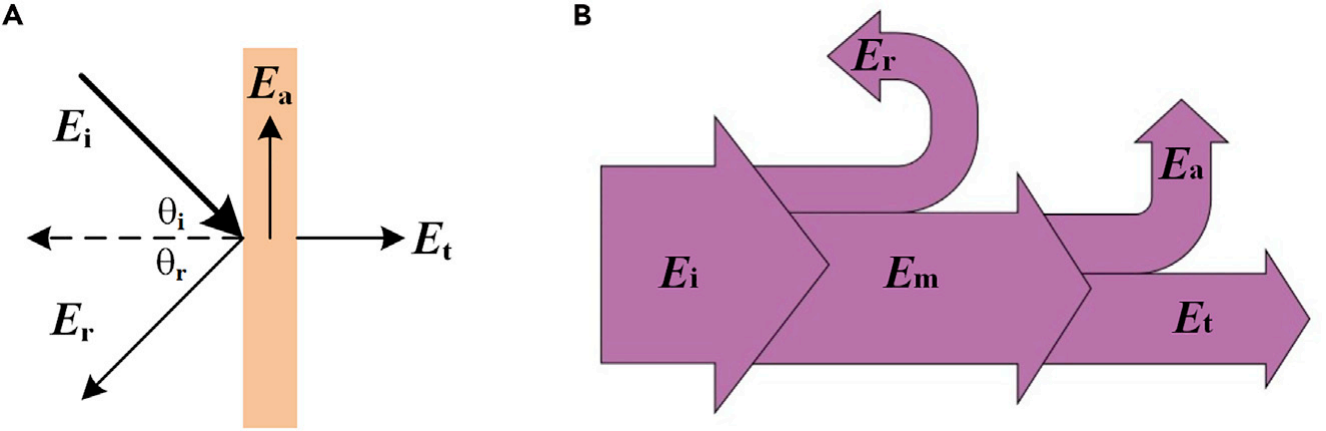
Sound Propagation (A) Sound propagation process. Adapted from (Bujoreanu et al., 2017), Copyright (2017), with permission from Elsevier. (B) Sound energy conservation.

When an incident acoustic wave strikes a material, as shown in Figures 2A and 2B, a portion of incident energy $E_{\text{i}}$ is reflected due to the impedance mismatch, and the reflected energy is denoted as $E_{\text{r}}$. The rest of $E_{\text{i}}$ enters the material, which is denoted as $E_{\text{m}}$. Then, the acoustic energy $E_{\text{m}}$ will transmit through the material (transmitted energy $E_{\text{t}}$) or be absorbed (absorbed energy $E_{\text{a}}$). According to the law of energy conservation

(Equation 1) $$E_{\text{i}}=E_{\text{r}}+E_{\text{a}}+E_{\text{t}}$$

The sound absorption coefficient α is an important parameter for evaluation of the sound absorption efficiency of a material, which is defined as the ratio of the absorbed energy to the incident energy:

(Equation 2) $$\alpha =E_{\text{a}}/E_{\text{i}}$$

However, it is difficult to measure the absorbed energy $E_{\text{a}}$ experimentally. Therefore, the sound absorption coefficient is usually calculated through reflected and transmitted energy indirectly by the following equation:

(Equation 3) $$\alpha =1-\left(E_{\text{r}}+E_{\text{t}}\right)/E_{\text{i}}$$

To describe the absorptive properties of a given structure in a specific bandwidth, a parameter $Q_{\alpha }$ was introduced as the average value of the sound absorption coefficient in a specific bandwidth by José Sánchez-Dehesa's group (Climente et al., 2012):

(Equation 4) $$Q_{\alpha }=\frac{1}{\Delta \nu }\int_{\nu_{i}}^{\nu_{f}}\alpha \left(\nu \right)\text{d}\nu$$

where $\Delta \nu =\nu_{f}-\nu_{i}$ is the bandwidth. The α and $Q_{\alpha }$ parameters are important to evaluate the sound absorption performance of AMs in the following introduction of airborne, waterborne, and gradient-index AMs.

## Airborne Sound Absorbing AMs

### Membrane-type AMs

**Figure 3:**
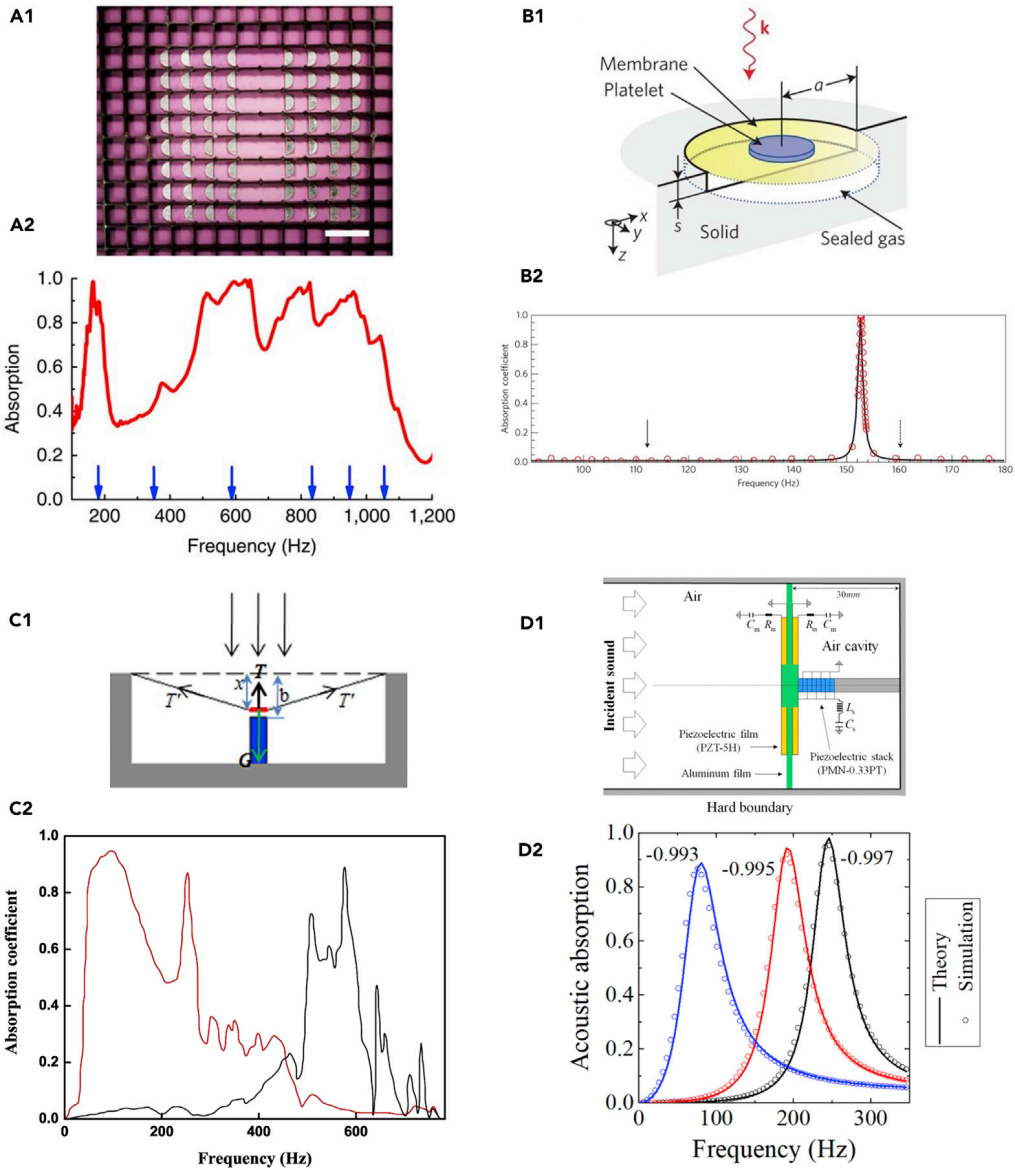
Membrane-type Sound Absorbing AMs (A) (A1) A sample photograph of dark AM. Scale bar, 30 mm. (A2) Experimentally measured absorption coefficients for two layers of the sample in (A1) with a reflector placed 28 mm behind the second layer. Absorption peaks were observed at 164, 376, 511, 645, 827, and 960 Hz. (A1 and A2) Reprinted with permission from (Mei et al., 2012): Springer Nature, Copyright (2012). (B) (B1) Schematic of a metasurface consisting of an elastic membrane decorated by a platelet, a reflecting surface, and a thin sealed gas layer in between. (B2) Measured absorption coefficients of the sample in (B1). The absorption peak, reaching 0.994, was observed at 152 Hz. (B1 and B2) Reprinted with permission from (Ma et al., 2014): Springer Nature, Copyright (2014). (C) (C1) Schematic of an AM absorber with magnetic negative stiffness. The surface of the membrane could be adjusted by the magnetic force and the membrane tension. (C2) The absorption peak of the sample in (C1) could be shifted to lower frequencies. The red line was obtained with negative stiffness, whereas the black line was obtained without negative stiffness. (C1 and C2) Reprinted with permission from (Zhao et al., 2017). Copyright (2017), Acoustic Society of America. (D) (D1) Schematic of adaptive AM. (D2) Sound absorption spectrum of adaptive AM in (D1) with the piezoelectric stack, in which the three capacitance values in the shunting circuit of the stack were 0.993, 0.995, and 0.997. (D1 and D2) Reprinted from (Liao et al., 2019), © IOP Publishing. Reproduced with permission. All rights reserved.

Membrane-type AMs are usually elastic membranes embedded within masses, which form spring-mass systems. Membrane-type AMs are considered, initially, reflective metamaterials for sound insulation (Yang et al., 2008, Yang et al., 2010, Zhang et al., 2013). To extend the absorption function of membrane-type AMs, Sheng's group proposed an absorber consisting of an elastic membrane decorated with asymmetric rigid platelets (Figure 3A1) (Mei et al., 2012). The proposed absorber could absorb sound at 100–1,000 Hz (Figure 3A2), and the corresponding sound wavelengths (340–3,400 mm) were three orders of magnitude larger than the membrane thickness (0.2 mm thick for elastic membrane and 1 mm thick for platelets). Furthermore, Sheng's group reported a metasurface consisting of an elastic membrane decorated by a platelet, a reflecting surface, and a thin sealed gas layer in between (Figure 3B1) (Ma et al., 2014). This metasurface can obtain perfect absorption at specific frequencies under 500 Hz (Figure 3B2), and the absorbed acoustic energy can be converted to electrical current. In their work, the observed high acoustic-electrical energy conversion efficiency was 23%. Furthermore, asymmetric AM structures could be employed to enhance sound absorption performance. In addition, there are also membrane-type AMs backed by a cavity. For an AM with a large back cavity, magnetic negative stiffness could be employed to reduce the back cavity size and shift the absorption peak to lower frequencies (Figures 3C1 and C2) (Zhao et al., 2017). Furthermore, adaptive stiffness of AMs could be employed to broaden low-frequency sound absorption (Figures 3D1 and D2) (Liao et al., 2019). In addition, the acoustic siphon effect could be employed to reduce the thickness of units of a membrane-type AM (Liu et al., 2019b).

### AMs with Solid Scatterers

AMs can be grouped into solid-solid, fluid-fluid, and solid-fluid composite systems (Pennec et al., 2010). For solid-fluid AMs, when characteristic structures (usually regular structures) of AMs are solid, AMs are considered to have consisted of solid scatterers embedded in a fluid matrix. Early research into AMs based on solid scatterers focused on the band gap, which could be used for sound attenuation in a specific frequency range. Local resonance for sound attenuation in AMs was a research hotspot (Hirsekorn, 2004, Liu et al., 2000) because the wavelength corresponding to strong sound attenuation bands was approximately two orders of magnitude smaller than that predicted by Bragg's theory. However, the sound absorption band of AMs based on local resonance is usually narrow. As the studies on AMs are broadened, the inherent losses arising from thermal and viscous effects in AMs are also applied to absorb acoustic energy directly and effectively. In José Sánchez-Dehesa's laboratory, AM absorbers based on multilayered sonic crystals were investigated by theoretical analyses, numerical simulations, and experiments considering thermoviscous losses (Guild et al., 2015). To enhance the AM sound absorption based on solid scatterers, the combination of AMs and other absorbing material could be effective and feasible. For example, embedding steel spheres into porous foam could improve low-frequency sound absorption (Slagle and Fuller, 2015). The effects of shapes of solid inclusions on sound absorption were further studied by Groby (Groby et al., 2014). Besides, added perforated shells for AMs could exhibit high absorption levels (Sanchez-Dehesa and Garcia-Chocano, 2015). There are additional specific studies into the factors influencing AM sound absorption performance, such as the crystal filling fraction, scatterer's coating layer, and type of backing (Christensen et al., 2014, Lu et al., 2011).

### AMs with Fluid Scatterers/Cavities

#### Fluid Scatterers

Corresponding to the solid scatterers mentioned in the section AMs with Solid Scatterers, AMs are considered to consist of fluid scatterers embedded in a solid matrix when characteristic AM structures (usually regular structures) are fluid. AMs with fluid scatterers were initially applied to sound insulation. Subsequently, AMs with fluid scatterers were also exploited for sound absorption. For example, Wen's group analyzed the absorption performances of an AM with an Alberich anechoic layer and backing (Meng et al., 2012a). Weisser (Weisser et al., 2016) proposed an AM with fluid scatterers surrounded by poroelastic host materials to broaden the efficient sound absorption band. Moreover, when the fluid inclusions were connected, the sound absorption band could be broadened (Matlack et al., 2016). When the second manifestation of porosity was introduced into a porous material (Sgard et al., 2005), the so-called double porosity AMs were found to be able to improve sound absorption (Cui and Harne, 2017). There were further studies that considered the effects of fluid inclusion shapes and compression on the acoustic properties of AMs (Overvelde et al., 2012, Shan et al., 2014).

#### Cavity-Based AMs

**Figure 4:**
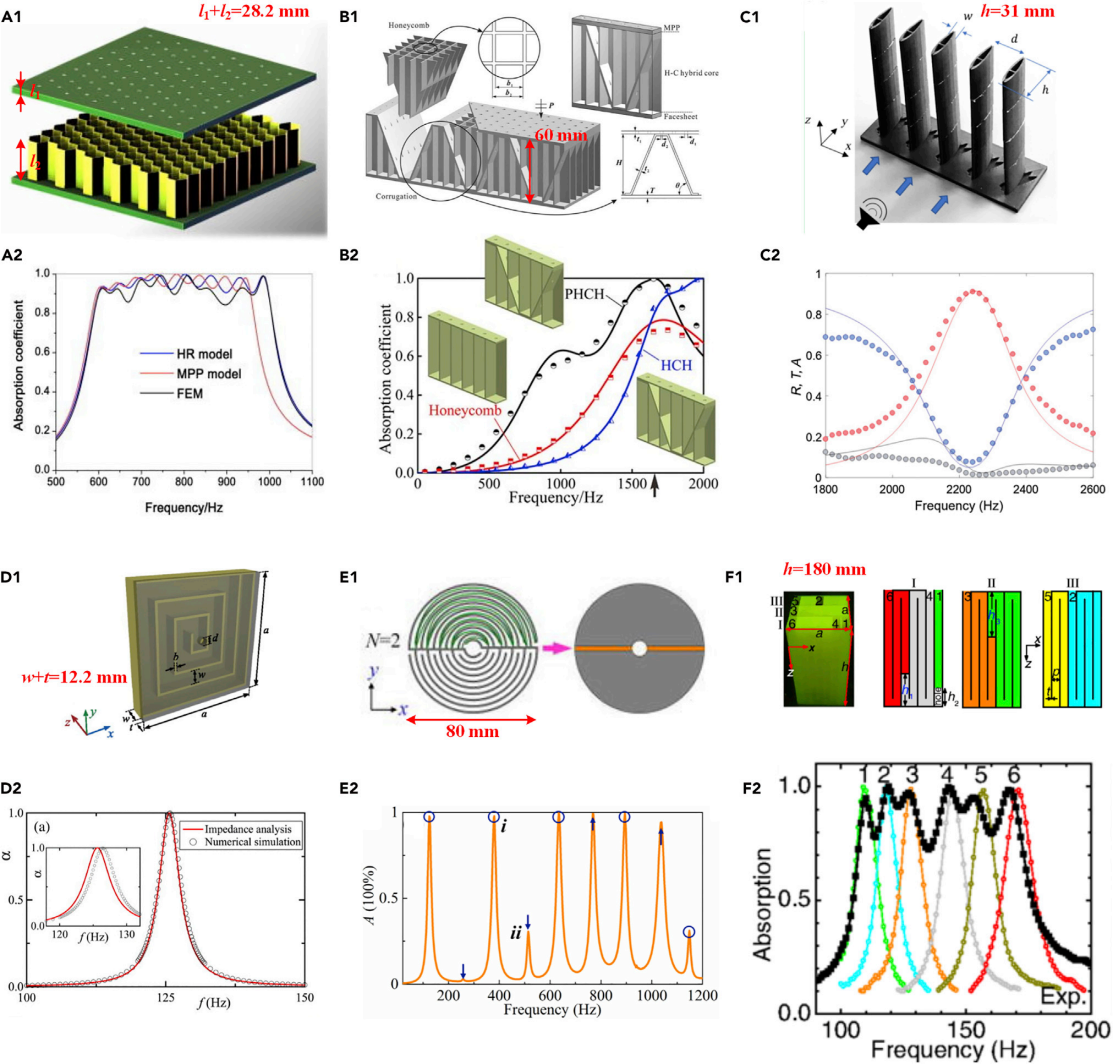
Cavity-Based Sound Absorbing AMs (A) (A1) Schematic of composite honeycomb absorber. The sample size along with incident wave was 28.2 mm. (A2) Sound absorption coefficients of the sample in (A1). (A1 and A2) Reprinted with permission from (Peng et al., 2018). Copyright (2018), Acoustic Society of America . (B) (B1) Schematic of perforated honeycomb-corrugated hybrid absorber, consisting of a micro-perforated panel, a honeycomb-corrugation hybrid core, and a panel. The sample size along with incident wave was 60 mm. (B2) Sound absorption coefficients of the sample (PHCH) in (B1) compared with competing structures (honeycomb and HCH). (B1 and B2) Reprinted from (Tang et al., 2017). (C1) An image of 3D-printed sparsely arranged acoustic absorber with fish shape, allowing ventilation. The sample size along with incident wave was 31 mm. (C2) Measured acoustic spectra for the sample shown in (C1). (C1 and C2) Reprinted with permission from (Lee et al., 2019), Copyright (2019) by the American Physical Society. (D) (D1) Schematic of the metasurface absorber consisting of a perforated plate (transparent gray region) with a hole and a coiled chamber. The sample size along with incident wave was 12.2 mm. (D2) Sound absorption coefficients of the sample in (D1). The absorption peak was realized at 125.8 Hz. (D1 and D2) Reprinted from (Li and Assouar, 2016), with the permission of AIP Publishing. (E) (E1) Schematic of a double-channel Mie resonator. The sample size along with incident wave was 80 mm. (E2) Sound absorption coefficients of the sample in (E1). Multiple absorption peaks were observed. (E1 and E2) Reprinted from (Long et al., 2018b), with the permission of AIP Publishing. (F) (F1) An image of the labyrinthine AM (left panel) and schematic illustration of the six channels (three right panels). The sample size along with incident wave was 180 mm. (F2) Measured absorption spectra of the sample shown in (F1). (F1 and F2) Reprinted with permission from (Zhang and Hu, 2016), Copyright (2016) by the American Physical Society.

Perforated panels have been widely used for sound absorbing materials/structures (Maa, 1998). Recently, metamaterial-based perforated panels have been proposed by combining a perforated host panel and acoustic metastructures. For example, a perforated host panel combined with spring-damper resonators was proposed to improve sound absorption below 1,000 Hz (Ren et al., 2019). A thin structured rigid body, which consisted of a perforated plate over a deep subwavelength narrow channel of air, had good sound absorption performance at around 12.6 kHz (Starkey et al., 2017). Low-frequency absorbers, which consisted of perforations over a honeycomb core, were proposed to enhance absorption performance (Figures 4A1 and A2) (Peng et al., 2018). A hybrid acoustic absorber, which consisted of a perforated panel and honeycomb-corrugation hybrid core, was proposed to absorb acoustic waves below 2,000 Hz (Figures 4B1 and B2) (Tang et al., 2017). A perforated panel with subcavities at different depths could achieve good sound absorption in the frequency range of 450–3,500 Hz (Min and Guo, 2019). An absorber based on a double-layered perforated metastructure could not only control noise but also allow ventilation (Li et al., 2018).

Besides, Helmholtz resonators are classical devices, which can be applied to enhance the absorption of acoustic energy (Romero-Garcia et al., 2016). For example, a Helmholtz resonator with embedded apertures for low-frequency perfect absorption was proposed, and its thickness was only approximately one-fiftieth of the operating wavelength (Huang et al., 2019a). A thin panel placed with one layer of Helmholtz resonators could be adopted to absorb sound at around 340 Hz (Jiménez et al., 2016). An AM composed of two 180°-twisted split tubes was proposed to absorb sound below 500 Hz (Wu et al., 2016). A similar study could be found in Gao et al. (2018). Local defect elements of AMs could also be applied to improve sound absorption (Yahya, 2017). An open Helmholtz resonator with sound absorption and sound delay performances was realized (Ma et al., 2020). Further studies on Helmholtz resonators for sound absorption include nonlinearity of a Helmholtz resonator (Achilleos et al., 2016) and a multi-Helmholtz resonator (Long et al., 2018a). In addition, Jimenez (Jimenez et al., 2017) emphasized the importance of breaking the symmetry of the system with Helmholtz resonators to achieve quasiperfect absorption, and the quasiperfect absorption was obtained at 350 Hz. In asymmetric structures for Helmholtz resonators, near-perfect absorption was achieved with a deep subwavelength sample in a two-port system with one-sided incident wave (Merkel et al., 2015). Furthermore, multiband and broadband absorption was achieved by asymmetric Helmholtz resonators (Long et al., 2017a), and corresponding theoretical, numerical, and experimental results verified that sound energy could be almost totally absorbed (96.1%) at 373 Hz. The optimization of distributions and shapes of units was presented to improve AM sound attenuation capabilities (Romero-Garcia et al., 2008, Romero-Garcia et al., 2009). The advantages of perforated panels and Helmholtz resonators can be combined as well. For example, an acoustic liner, which consisted of a perforated panel over a honeycomb liner with an array of Helmholtz resonators, was proposed to improve low-frequency noise reduction (Beck et al., 2015). To obtain broadband absorption, an AM consisting of a Helmholtz resonator with inserted perforated composite was proposed to exhibit near-perfect absorption in the range 450–1,360 Hz (Liu et al., 2019a). In addition, Fabry-Perot resonators were also exploited for sound absorption, and additional details on Fabry-Perot resonances could be found in Estrada et al. (2012) and Luna-Acosta and Makarov (2009).

Furthermore, the cavities in the above-mentioned perforated panels and Helmholtz resonators can be abstracted away to design sound absorbing AMs. Specifically, the absorption of an AM can be enhanced by the addition of microslits (Ruiz et al., 2016). Steel tubes with periodic narrow slits were proposed to attenuate sound in the low-frequency audible range (approximately 800 Hz to 10 kHz) (Cui et al., 2008). Similarly, a slim absorber with coupled microslits was proposed to attenuate sound below 1,000 Hz (Zhao et al., 2018). A further multislit absorber, which introduced microslit structures into a mesoslit matrix, was proposed to absorb acoustic waves over a wide frequency range (Ren et al., 2016). Besides, to allow fluid flow and light propagation, the fish-shaped units with absorber silts were sparsely distributed, and the sound absorption coefficient reached a peak at approximately 2,220 Hz (Figures 4C1 and C2) (Lee et al., 2019). After a structure consisting of a periodic arrangement of narrow slits was studied, Groby (Groby et al., 2015) showed that slow sound propagation associated with its inherent dissipation could be adopted to design sound absorbing materials. Then, Fernández-Marín (Fernández-Marín et al., 2019) proposed that a building block consisting of cavity-backed aerogel clamped plates would perfectly absorb sound at around 600 Hz.

For sound absorption and space-saving purposes, coiling the AM structures is an efficient method to reduce the scale because the physical dimensions of the structures are substantially shorter than the coiled path. For example, sound absorbing panels with coiled tubes were proposed to perfectly absorb sound at around 400 Hz (Cai et al., 2014). Then, Li and Assouar presented a similar absorber that could absorb sound at around 125 Hz (Figures 4D1 and D2) (Li and Assouar, 2016). Subsequently, Li's group (Huang et al., 2018) designed similar structures to achieve perfect absorption at around 146.75, 158, and 168 Hz. However, the above-mentioned sound absorbers exhibited good sound absorption only at a single frequency. Therefore, to broaden the sound absorption range, Wen's group (Wang et al., 2017) proposed that two different coiled structures could be combined. Similar verification could be found in Ryoo and Jeon (2018). Spiderweb-inspired structures can provide broadband sound absorption as well (Figures 4E1 and E2) (Long et al., 2018b). Subsequently, an absorber with underdamped coiled space resonators was designed by the causality principle to achieve broadband near-perfect absorption (Long et al., 2019), and the frequency regime of near-perfect absorptance (at 95%) was obtained from 228 to 319 Hz. Besides, multiple internal reflections could be harnessed to achieve highly absorptive AMs (Shen and Cummer, 2018). In general, it is the lengthened paths or multiple resonant cavities that lead to enhanced dissipation in the structure-coiled absorbers (Elayouch et al., 2018). More complicated paths require labyrinthine AMs. For example, a 3D single-port labyrinthine AM was proposed to perfectly absorb airborne sound at a frequency ranging from 100 to 200 Hz (Figures 4F1 and F2) (Zhang and Hu, 2016). Subsequently, Hu's group (Liu et al., 2017) further demonstrated that, by introducing porous media (such as cotton) inside labyrinthine channels, the sound absorption could be enhanced. A similar labyrinthine AM with 10.86-cm-thick structure was designed by causality constraint to obtain broadband absorption spectrum (Yang et al., 2017). Moreover, introducing multichannels in labyrinthine AM was another way to enhance sound absorption (Chang et al., 2018).

In addition, sound absorption AMs, which exploit cavities, include fractal/hierarchical structures (Miniaci et al., 2018, Mousanezhad et al., 2015, Song et al., 2016), symmetrical and anti-symmetrical structures (Long et al., 2017b, Wei et al., 2014), asymmetric structures (Merkel et al., 2015), bio-inspired double resonators (Huang et al., 2019b), multiresonators (Barnhart et al., 2019), degenerate resonators (Piper et al., 2014, Yang et al., 2015), and non-Hermitian AMs (Achilleos et al., 2017). An overview of typical cavity-based AMs for sound absorption is listed in Table 1.

**Table 1:** Typical Cavity-Based AMs for Sound Absorption

Structures	Maximum Shape Size along with Incident Wave (mm)	Research Type	Function	Effective Frequency Range	Ref.
A lightweight sandwich plate with honeycomb-corrugation hybrid core	60	Ana, Sim	Absorption	Below 2,000 Hz	Tang et al., 2017
A hollow pipe attached by eight double-layered perforated metastructures	54.5	Ana, Exp, Sim	Noise control and ventilation	800–1,000 Hz	Li et al., 2018
Helmholtz resonators with embedded apertures	50	Ana, Exp	Absorption	130–170 Hz	Huang et al., 2019a
Helmholtz resonator with inserted perforated composite	62	Ana, Exp, Sim	Absorption	450–1,360 Hz	Liu et al., 2019a
Sparsely distributed resonators fish shape	31	Ana, Exp, Sim	Noise control and allowing ventilation	2,100–2,300 Hz	Lee et al., 2019
A building block consisting of cavity backed aerogel clamped plates	42	Ana, Exp, Sim	Absorption	Around 600 Hz	Fernández-Marín et al., 2019
Panel comprising coiled coplanar tubes	17	Exp, Sim	Absorption	Around 600 Hz	Cai et al., 2014
Coiled channels with embedded apertures	24, 21.5, and 20	Ana, Exp	Absorption	Around 146.75 Hz, 158 Hz, and 168 Hz	Huang et al., 2018
Double-channel Mie resonator	80	Exp, Sim	Absorption	100–1,100 Hz	Long et al., 2018b
Multiple and neighboring resonant cavities	193.5	Exp, Sim	Absorption	200-400 Hz, and 1,000-1,350 Hz	Elayouch et al., 2018
Lumped-mass inclusions embedded in a poroelastic matrix (polyurethane foam)	50.8	Exp, Sim	Trapping and attenuating acoustic energy	600-1,600 Hz	Harne et al., 2017

Ana, Exp, and Sim represent analysis, experiment, and simulation, respectively.

## Waterborne Sound Absorbing AMs

**Figure 5:**
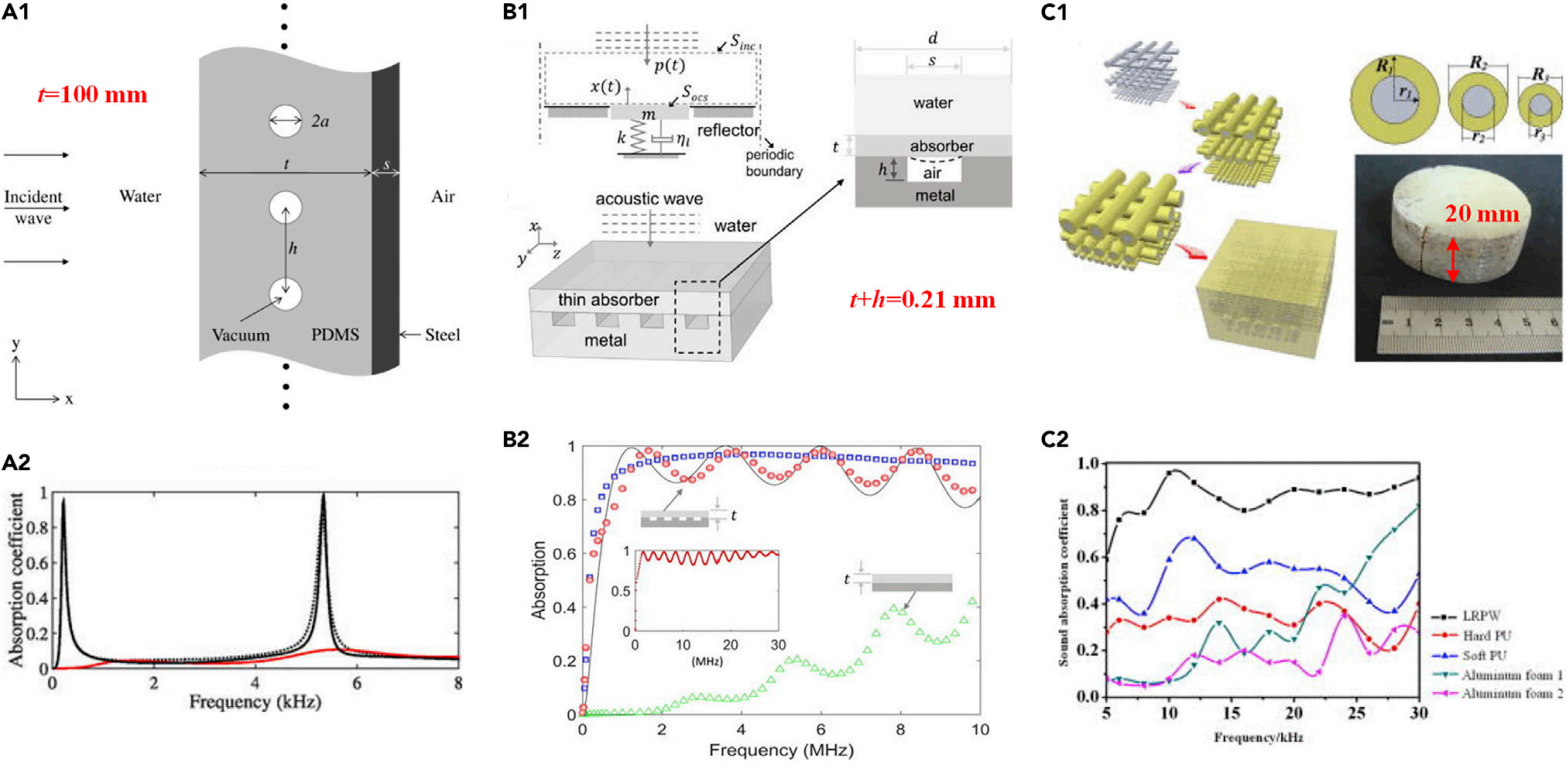
Underwater Sound Absorbing AMs (A) (A1) Schematic of periodic cylindrical cavities embedded in a polydimethylsiloxane (PDMS) medium. The thickness along with incident wave was *t* = 100 mm. (A2) Analytical (black solid line) and numerical (black dotted line) absorption coefficients of the sample in (A1) compared with unvoided sample (red line). (A1 and A2) Reprinted with permission from (Sharma et al., 2017). Copyright (2017), Acoustic Society of America. (B) (B1) Schematic of an acoustic metasurface consisting of a metal grating covered with a PDMS layer. The thickness along with incident acoustic wave was 0.21 mm. (B2) Analytical (solid line) and numerical (red circle) acoustic absorption spectra of the sample in (B1) compared with planar sample with no metal grating (green triangle). (B1 and B2) Reprinted from (Lee and Iizuka, 2018), with the permission of AIP Publishing. (C) (C1) Schematic and image of LRPW. The thickness of the sample was 20 mm. (C2) Absorption coefficients of the sample in (C1) compared with those of other underwater materials. (C1 and C2) Reprinted from (Jiang et al., 2009), with the permission of AIP Publishing.

Waterborne sound absorbing materials are urgently needed in underwater acoustic communication system, sonar evasion, and other applications. However, research on waterborne sound absorbing materials progresses slowly due to the complex marine environment and high hydrostatic pressure. Benefiting from AMs, waterborne sound absorbing materials face new development. For example, Lanoy (Leroy et al., 2015) demonstrated an AM with air bubbles for sound absorption, and the high absorption (higher than 91%) was obtained over the 1.4–2.9 MHz range. Ivansson proposed that air-filled cavities covered by rubber coating could be used as anechoic submarine coatings (Ivansson, 2006) and conducted corresponding numerical design and analysis (Ivansson, 2008). Similarly, Sharma (Sharma et al., 2017) presented an AM comprising periodic voids in a soft elastic medium attached to a steel backing (Figures 5A1 and A2). Another study on the anechoic properties of air spheres in rubber was performed by Wen's group (Zhao et al., 2007). Besides, high-density spheres coated by viscoelastic matrix are an effective method for waterborne sound absorption. Moreover, Wen's group conducted further analysis (Huang et al., 2016) and optimization (Meng et al., 2012b) targeting high-density spheres coated by a viscoelastic matrix. Wen's group (Yuan et al., 2013) also demonstrated that by softening coats of resonant units, the starting frequency of Bragg scattering shifted to the subwavelength region when the matrix was a low-shear-velocity medium. However, the underwater AMs with a single coating layer usually possess only relatively narrow sound absorption bands. To overcome that drawback, an AM with multiple coating layers was proposed by Shi (Shi et al., 2019) and an AM with different scatterer sizes was proposed by Zhang (Zhang et al., 2018).

Recently, an acoustic metasurface, consisting of a metal grating covered with a polydimethylsiloxane layer, was proposed and exhibited high absorption over 0.5–10 MHz (Figures 5B1 and B2) (Lee and Iizuka, 2018). Topological structures of acoustic metasurfaces were investigated by Lee (Lee et al., 2018). Besides, inspired by woodpile photonic crystals, a locally resonant phononic woodpile (LRPW) was proposed by Jiang (Jiang et al., 2009) to enhance the underwater sound absorption in a wide frequency range (Figures 5C1 and C2). The LRPW consisted of steel rods with three different sizes, soft polyurethane (PU), and hard PU. The LRPW was proved to possess strong underwater acoustic absorbance with the absorption coefficient over 0.8 from 8 to 30 kHz. After LRPW, Jiang (Jiang et al., 2010) also proposed a composite structure that combined the locally resonant PC and interpenetrating network structure to achieve a broadband underwater strong acoustic absorption.

## Gradient-Index Sound Absorbing AMs

Although AMs impart extraordinary properties, most of those properties are usually single frequency or narrow band because high absorption would occur only in the vicinity of resonant frequencies. To overcome that limit, gradient-index structures for AMs are proposed. Gradient-index AMs can be achieved by modulating the characteristic factors, such as the radii or elastic properties of the inclusions, the lattice spacing or width, orientation angle, and thickness. The first gradient-index AMs were proposed for sound focusing (Torrent and Sánchez-Dehesa, 2007). Subsequently, gradient-index AMs have attracted considerable attention to broaden the band of acoustic properties such as sound insulation and absorption.

**Figure 6:**
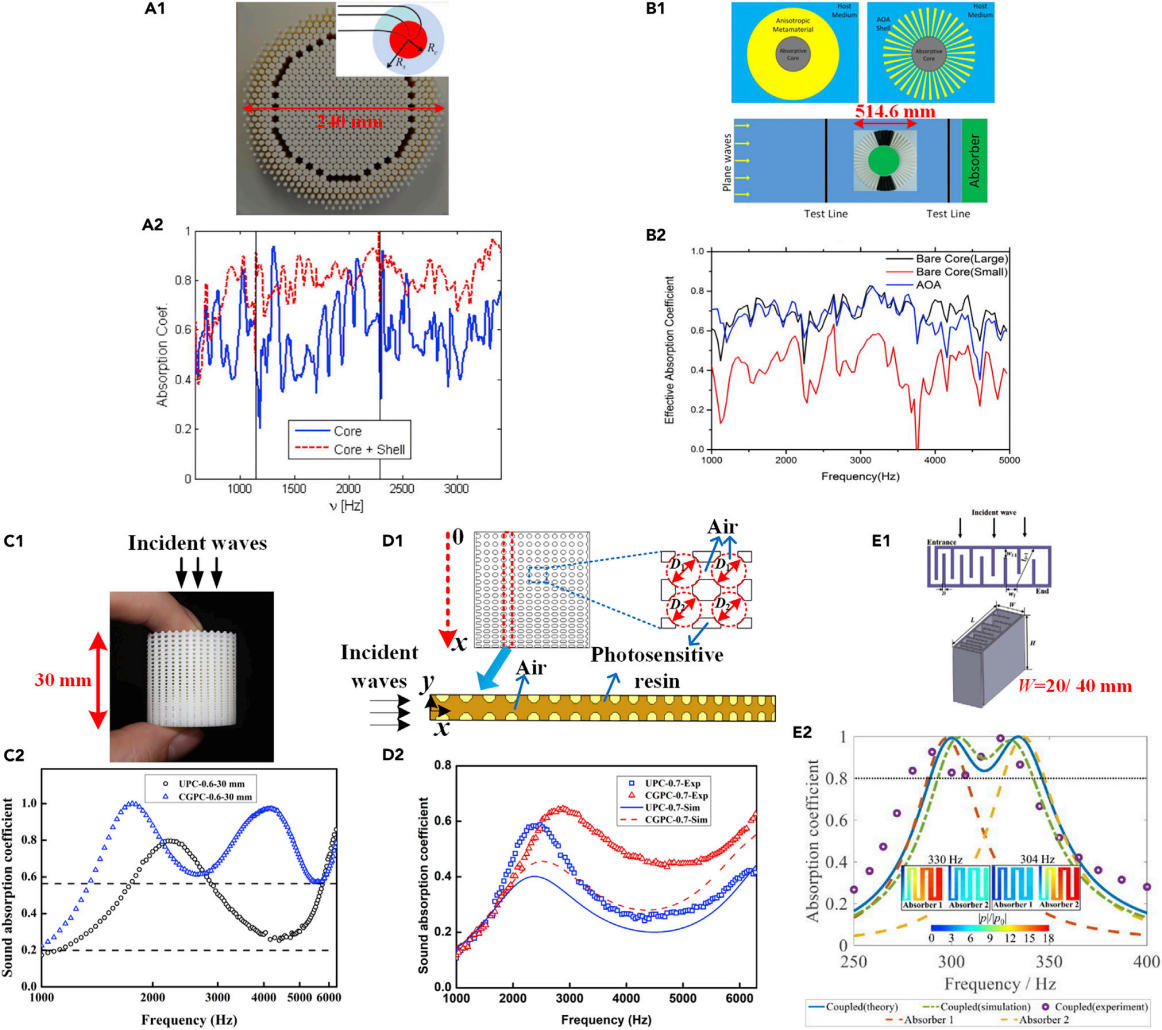
Gradient-Index Sound Absorbing AMs (A) (A1) Image of a novel acoustic omnidirectional absorber consisting of a shell and a core. The outer shell was composed of cylinders with different diameters, and the diameters increased with decreasing distance to the center. The maximum diameter of above sample was 240 mm. (A2) Measured absorption coefficients of the sample in (A1) compared with a reference sample with only core (continuous blue line). (A1 and A2) Reprinted from (Climente et al., 2012), with the permission of AIP Publishing. (B) (B1) Schematic and image of an acoustic omnidirectional absorber in which the shell was simplified and composed of trapezoidal acrylonitrile butadiene styrene fins. The maximum diameter of the above sample was 514.6 mm. (B2) The measured effective absorption coefficients. The absorption property of the sample in (B1) could mimic a large absorptive core almost perfectly. (B1 and B2) Reprinted from (Gu et al., 2015), with the permission of AIP Publishing. (C) (C1) Image of sample CGPC-0.6-30 mm consisting of decreased pores along with incident wave. The sample thickness along with incident wave was 30 mm. (C2) Measured sound absorption coefficients of the sample in (C1) compared with uniform phononic crystal (UPC-0.6-30 mm). Sample CGPC-0.6-30 mm possessed high sound absorption coefficients (higher than 0.56) ranging from 1,350 to 6,300 Hz. (C1 and C2) Adapted from (Zhang et al., 2016). (D) (D1) Cross-sectional schematic of CGPC-0.7, and geometric model for the simulation. (D2) Experimental and simulated results of sound absorption coefficients of CGPC-0.7. Exp and Sim represent experimental and simulated results, respectively. (D1 and D2) Adapted from (Zhang et al., 2019b), Copyright (2019) The Japan Society of Applied Physics. (E) (E1) Schematic structure of a gradient cross-section absorber. The width *W* was chosen as 20/40 mm. (E2) Absorption spectra of two individual absorbers with L×W×H=30 mm×40 mm×50 mm. Two absorption peaks were observed. (E1 and E2) Reprinted from (Shen et al., 2019), with the permission of AIP Publishing.

When it comes to sound absorption of a material, gradient-index structures can bring broadband property as well. For an absorbing material based on the dissipative effect, there are two important conditions for excellent sound absorption: superior dissipation capacity within the material and little reflection at the fluid-structure interface. Therefore, no matter how the dissipation of the material capacity is, the sound absorption can be weak if significant reflection occurs at the fluid-structure interface. Hence, impedance matching is important in sound absorption. Therefore, a gradient-index structure is an effective method to improve sound absorption performance. For example, José Sánchez-Dehesa's group designed a novel acoustic omnidirectional absorber consisting of a shell and a core, as shown in Figures 6A1 and A2 (Climente et al., 2012). The outer shell was composed of cylinders with different diameters, and the diameters increased with decreasing distance to the center. The gradient structures of the shell matched the acoustic impedance of the air and the designed acoustic omnidirectional absorber. Then, the shell served as a gradient-index lens to focus the acoustic wave into the core in which acoustic energy would be dissipated. The absorptive property of the acoustic omnidirectional absorber was enhanced by approximately 20% compared with that of only the core of the above-mentioned absorber through the self-defined evaluation parameter $Q_{\alpha }$ in Equation (4). To simplify the shell, Cheng's group designed a similar acoustic omnidirectional absorber in which the shell was composed of trapezoidal acrylonitrile butadiene styrene fins (Figures 6B1 and B2) (Gu et al., 2015). The acoustic omnidirectional absorber with fin-like shell also exhibited enhanced absorption when compared with that of the small bare core. Subsequently, the acoustic omnidirectional absorber with a guiding shell and a dissipated core was extended to underwater operation (Naify et al., 2014). To reduce the size of the absorber, a 3D continuously graded phononic crystal (CGPC) without an outer shell was proposed (Figure 6C1) (Zhang et al., 2016, Zhang et al., 2019a). The thickness of CGPC was only 30 mm, which was clearly smaller than 240 mm (the diameter in Figure 6A1) or 514.6 mm (the diameter in Figure 6B1). The proposed CGPC consisted of gradient-index interpenetrating pores in the absorption core directly instead of the additional gradient-index shell mentioned in Climente et al. (2012). The corresponding experimental results showed that the sound absorption coefficients of a CGPC with a porosity of 0.6 were higher than 0.56 when the frequency was 1,350–6,300 Hz (Figure 6C2). To simplify the calculation model, a 2D CGPC was further proposed (Figure 6D1) (Zhang et al., 2019b) and simulated with consideration of viscous and thermal effects. The predicted sound absorption coefficients of CGPC-0.7 were qualitatively verified by experimentally measured results, as shown in Figure 6D2. To achieve sound wave attenuation of frequencies below 500 Hz, in-uniform gradient cross-section (GCS) channels were proposed, which consisted of coiled channels of discrete GCSs (Figure 6E1) (Shen et al., 2019). When two GCS absorbers were combined, relatively broadened absorption spectra could be obtained (Figure 6E2). Gradient-index AMs for sound absorption with experimental evidence are listed in Table 2.

**Table 2:** Gradient-Index AMs for Sound Absorption with Experimental Evidence

Method for Achieving Gradient-Index Structures	Material of Inclusions	Material of Matrix	Maximum Shape Size along with Incident Wave (mm)	Function	Ref.
Modulating radii of the inclusions	Plastic	Air	240	Focusing the acoustic wave into the dissipated core	Climente et al., 2012
Fin-like structure	Acrylonitrile butadiene styrene	Air	514.6	Focusing the acoustic wave into the dissipated core	Gu et al., 2015
Modulating radii of the inclusions	Rubber	Water	356	Focusing the acoustic wave into the dissipated core	Naify et al., 2014
Modulating radii of the inclusions	Aluminum rod	Air	1,480	Improving sound absorption	Elliott et al., 2014
Modulating radii of the inclusions	Air	Photosensitive resin	30	Broadening and improving sound absorption	Zhang et al., 2016, Zhang et al., 2019a
Modulating cross-section	Polylactide	Air	40	Broadening sound absorption	Shen et al., 2019
Modulating thicknesses of the porous layer	Porous material	Air	70	Broadening and improving sound absorption	Fang et al., 2018a, Fang et al., 2018b
Modulating height of the sawtooth	Thermoplastics	Air	370	Broadening sound absorption	Jiang et al., 2014

In general, gradient-index structures can be applied to broaden and improve the sound absorption performance of AMs. The underlying reasons for the above-mentioned broadened band and improvement come down to three points: impedance matching, acoustic energy loss within the gradient-index structures, and propagation path. Good impedance matching at the AM fluid-structure interface results in little reflection, which affects the maximum value of the sound absorption coefficient. Among the acoustic energy loss, viscous and thermal dissipation within the AMs cannot be neglected. The structures of AMs have an impact on the propagation path of acoustic waves, which has a close connection to the viscous and thermal dissipation within the AMs. Besides, it should be noted that, when the AMs are in water instead of air, thermal effects could be neglected to simplify the simulation due to the low thermal expansion coefficient.

## Summary and Perspective

The last three decades have witnessed tremendous developments in the classical field of acoustics thanks to the emergence of AMs and new fabrication technologies (such as 3D printing). In this review, the basic concepts for AM sound absorption are introduced so that the review is suitable for beginners as well as experts in field of AMs. The sound absorption of AMs is classified, based on the background media, into airborne and waterborne sound absorption. For airborne sound absorption, membrane-type AMs, AMs with solid scatterers, and AMs with fluid scatterers/cavities are systematically reviewed. Therein, cavity-based AMs (from traditional perforated panels and Helmholtz resonators to novel coiled and labyrinthine structures) are the focus because of their potential application for sound absorption. The AMs for waterborne sound absorption are also reviewed. The gradient-index AMs are singled out as a section because of their impedance matching, which can enhance the sound absorption.

**Figure 7:**
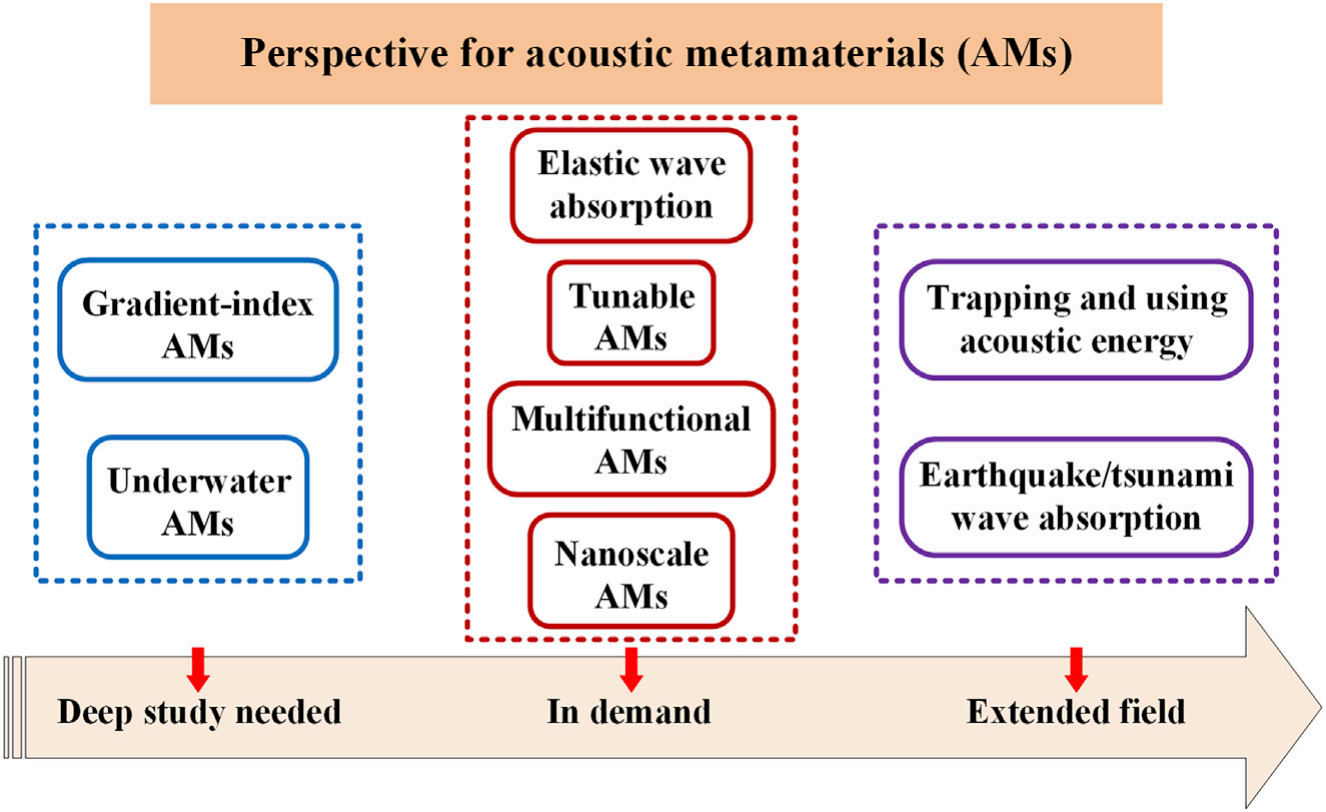
Perspective for Acoustic Metamaterials

The overall perspective for AMs is summarized in Figure 7. Initially, the three main challenges in sound absorption are the broad working band, low-frequency absorption, and effective absorption using small size. Although the reviewed studies have contributed to the three main challenges, there is still room for improvement targeting the three main challenges. **Gradient-index AMs**: Owing to good impedance matching and acoustic energy dissipation within the gradient-index structures, gradient-index AMs are promising next-generation sound absorbing materials, which can enhance the sound absorption performance compared with common uniform-index AMs. The design and optimization of gradient-index structures for particular applications still need specific in-depth studies. On the other hand, the sound dissipation in complicated gradient-index structures could be further improved. Besides, the study on the topology of AMs may contribute to further illumination of the underlying mechanism of AM sound absorption. **Underwater AMs:** Although the underwater experiments are more difficult and complicated than airborne experiments, underwater AMs are in great demand for anechoic coating of underwater vehicles.

Besides, most of the reviewed studies are only successful in the laboratory or in the initial stages of development. Although a company named Acoustic Metamaterials Group has started to develop metamaterials noise control technology, significant work remains to bring successful ideas (in a laboratory) into real-world applications because of the demand for adjustability, multifunctionality, and small size. **Elastic wave absorption:** Previous work on elastic wave absorption should be continued with a consideration of the widespread presence of elastic waves (such as Lamb wave and Rayleigh wave). **Tunable AMs**: An AM usually possesses a specific structure, and it is used for a specific application. Tunable AMs have the potential to be adopted for different applications by tuning the properties. The AMs may also be required to be tunable because of the subjectivity of the sound event perception. **Multifunctional AMs**: In modern society, multiple functions are expected to be integrated into one device to save space and cost. For example, AMs with a combination of absorption and vibration suppression, absorption, and ventilation are in demand. However, those studies are just in the initial stages, and more in-depth investigations are expected. **Nanoscale AMs:** Nanoscale heat transport and nanoelectronics have important roles in nanoscale devices. Nanoscale AMs have the potential to reduce the amount of heat transport to ultralow levels.

Moreover, AM sound absorption can be broadened to save energy and reduce the damage caused by the power of nature. **Trapping and using acoustic energy:** Theoretically, the acoustic energy in unwanted sound (such as noise) can be trapped, stored, and then exploited. The unwanted sound could be absorbed while the reuse of acoustic energy contributes to energy conservation. There are studies on the trapping of acoustic energy in which the absorbed acoustic energy is demonstrated to be converted to electrical current (Ma et al., 2014). The reuse of acoustic energy needs further investigation. Power generation systems based on the reuse of acoustic energy are exciting and expected. **Earthquake/tsunami wave absorption:** Although some countries, such as Japan and China, have earthquake/tsunami warning capability, attenuation of earthquake/tsunami wave is in demand to further reduce earthquake/tsunami damage.
